# Resistance Exercise Reduces Skeletal Muscle Cachexia and Improves Muscle Function in Rheumatoid Arthritis

**DOI:** 10.1155/2011/205691

**Published:** 2011-12-10

**Authors:** Salaheddin Sharif, James M. Thomas, David A. Donley, Diana L. Gilleland, Daniel E. Bonner, Jean L. McCrory, W. Guyton Hornsby, Hua Zhao, Mathew W. Lively, Jo Ann A. Hornsby, Stephen E. Alway

**Affiliations:** ^1^Human Performance Laboratory, West Virginia University School of Medicine, Morgantown, WV 26506-9227, USA; ^2^Laboratory of Muscle Biology and Sarcopenia, West Virginia University School of Medicine, Morgantown, WV 26506-9227, USA; ^3^Division of Exercise Physiology, Center for Cardiovascular and Respiratory Sciences, West Virginia University School of Medicine, Morgantown, WV 26506-9227, USA; ^4^Department of Medicine, West Virginia University School of Medicine, Morgantown, WV 26506-9227, USA; ^5^Division of Exercise Physiology, Robert C. Byrd Health Sciences Center, West Virginia University School of Medicine, 1 Medical Center, P.O. Box 9227, Morgantown, WV 26506-9227, USA

## Abstract

Rheumatoid arthritis (RA) is a chronic, systemic, autoimmune, inflammatory disease associated with cachexia (reduced muscle and increased fat). Although strength-training exercise has been used in persons with RA, it is not clear if it is effective for reducing cachexia. A 46-year-old woman was studied to determine: (i) if resistance exercise could reverse cachexia by improving muscle mass, fiber cross-sectional area, and muscle function; and (2) if elevated apoptotic signaling was involved in cachexia with RA and could be reduced by resistance training. A needle biopsy was obtained from the vastus lateralis muscle of the RA subject before and after 16 weeks of resistance training. Knee extensor strength increased by 13.6% and fatigue decreased by 2.8% Muscle mass increased by 2.1%. Average muscle fiber cross-sectional area increased by 49.7%, and muscle nuclei increased slightly after strength training from 0.08 to 0.12 nuclei/**μ**m^2^. In addition, there was a slight decrease (1.6%) in the number of apoptotic muscle nuclei after resistance training. This case study suggests that resistance training may be a good tool for increasing the number of nuclei per fiber area, decreasing apoptotic nuclei, and inducing fiber hypertrophy in persons with RA, thereby slowing or reversing rheumatoid cachexia.

## 1. Introduction

Rheumatoid arthritis (RA) is a chronic, systemic, autoimmune, inflammatory condition that is associated with a reduced life expectancy [1, 2]. Patients with RA have restricted range of motion of joints, joints pain and reduced physical functioning, increased resting energy expenditure, and higher mortality rate. In addition to the direct effects of RA on joints, this disease is characterized by a loss of muscle mass (rheumatoid cachexia), an increase in fat mass, and normal or high body mass index (BMI). More than 50% of RA patients develop rheumatoid cachexia and as a result lose muscle strength, and power, and reduce their levels of physical activity [3].

Habitual physical activity is decreased in RA patients due to joint pain, restricted mobility, fatigue, reduced muscle mass, strength, and endurance, and these will result in decreased physical fitness. RA-induced reductions in physical activity can result in muscle loss, which further exacerbates rheumatoid cachexia [4]. Furthermore, low physical activity increases the risk for RA patients to develop cardiovascular disease, osteoporosis, insulin resistance, and obesity [3].

Rheumatoid cachexia predominantly affects type II fibers, although both fiber types are susceptible to atrophy in RA [5]. Resistance exercise is a potential therapeutic approach to improve muscle mass, especially in type II fibers [6]; however, there is little consensus in the literature to support prescribing resistance or aerobic exercise in patients with RA. While exercise-induced improvements in muscle strength, physical function, and aerobic capacity have been reported in patients with RA (reviewed in [7]), it is not clear to what extent aerobic or strength (resistance) training might reduce rheumatoid cachexia, cardiovascular dysfunction, and cardiovascular risk factors and/or improve the quality of life of these patients.

The elevated production of cytokines (i.e., TNF-*α* and IL-1*β*) contributes to rheumatoid cachexia. Cytokines are among the important mechanisms that trigger cell death by apoptosis in many diseases including rheumatoid arthritis (reviewed in [8]). Apoptosis (nuclear apoptosis) is unique in skeletal muscle, because death of a myonucleus via apoptosis can occur without death of the entire cell. Nuclear apoptosis has been reported during skeletal muscle loss that is associated with aging, denervation, cardiovascular disease, and cancer [9–11]. While apoptosis in skeletal muscle can be triggered by cytokines [9], it is not known if nuclear apoptosis also contributes to muscle loss in rheumatoid cachexia. Furthermore, it is not clear if exercise might reduce proapoptotic signaling and improve muscle function in patients with RA. This information is critical for understanding the etiology of RA more completely and to determine the potential effects that exercise might have on rheumatoid cachexia. In this case study, we examine the effects of strength training on muscle structure and function, and nuclear apoptosis in a subject with RA.

## 2. Case Report

### 2.1. Subject Characteristics

A 46-year-old female was recruited from the outpatient Rheumatology Clinic at Ruby Memorial Hospital, Morgantown, WVa, USA. The subject was diagnosed with RA for 2 years according to the guidelines described by the American College of Rheumatology [12], and she had history of seasonal asthma and gastritis. She was treated with etanercept subcutaneously (50 mg/week), methotrexate (15 mg/week), and etodolac (300 mg/twice daily). The subject gave written informed consent to participate in this study and to conduct strength training.

### 2.2. Assessments of Disease Progression

The subject underwent a full physical examination and 12 lead ECG to rule out overt cardiovascular disease or any contraindication to strength training. Disease progression was assessed in 28 joints by the Disease Activity Score (DAS28) [13], and systemic inflammation was estimated by erythrocyte sedimentation rate (ESR) and fasting serum levels of C-reactive protein. Additional indices of general systemic cardiovascular risk included blood levels of triglycerides, total cholesterol, high-density lipoproteins (HDL), low- density lipoproteins (LDL), and very-low-density lipoproteins (VLDL).

### 2.3. Physical Characteristics

The subject's body mass index (BMI) was calculated from height and weight measurements that were made with the subject wearing a swimsuit and without shoes using a calibrated electrical scale. Resting energy expenditure (REE) was estimated using an open-circuit ventilated hood system indirect calorimetric method for 30 min under thermoneutral conditions in the fasting state (True Max 2400, ParvoMedics, Salt Lake City, Utah, USA). Fat mass and percent body fat were measured by 7-site skinfolds assessment, using a Harpenden skinfold caliper (British indicators, UK). Each skinfold site was assessed a minimum of two times. If measurements were not within 1 mm, a third measure was taken. Fat-free mass (FFM) was calculated as total bodyweight minus fat mass FM.

### 2.4. Functional and Cardiovascular Assessments

A 50-foot walking test and a 30s maximal sit-to-stand test were performed [14, 15]. Peak oxygen consumption (VO_2peak_) were measured using indirect calorimetry (True Max 2400, ParvoMedics, Salt Lake City, Utah,USA) during a graded treadmill exercise test at 2.5 m/h. The elevation of the treadmill was increased by one percent every minute. The graded exercise test was continued until the subject could no longer exercise. VO_2peak_ was established if the following criteria were met: (a) peak respiratory exchange ratio >1.10, (b) attainment of at least 85% of maximal heart rate and (c) ratings of perceived exertion (RPE) were 17 using the Borg 6–20 scale. 

### 2.5. Muscle Function

Knee extensor and flexor isokinetic torque was assessed on the subject's dominant limb. The measurements were obtained before and 24 hours after the subject's last exercise session using methods previously reported by our laboratory [14–16]. Torque was measured on a Biodex System 4 dynamometer (Biodex Inc., Shirley, NY, USA). Briefly, the subject sat with the hip joint flexed to 90°. The subject's trunk and pelvis were secured with nylon straps to prevent extraneous movement. Torque was recorded as the best of five maximal voluntary contractions on the dominant leg while the subject was seated, with velocities of contraction of 90 degrees/second.

### 2.6. Muscle Structural Characteristics

 A needle biopsy was obtained from the vastus lateralis muscle midway between the patella and the greater trochanter, at the anterior border of the iliotibial band. The tissue was frozen in liquid nitrogen and stored at −80°C. Frozen 10 *μ*m serial cross-sections were cut by at −20°C on a microtome. Average muscle fiber cross-sectional area (CSA) was determined by plainmetry from tissue sections with the basal lamina stained by immunocytochemistry (Developmental Studies Hybridoma Bank, Iowa City, Iowa, USA). CSA for type I and type II muscle fibers was then determined by planimetry from tissue cross-sections that had been incubated with mouse anti-F59 monoclonal antibody against type II myosin (2E8, Developmental Studies Hybridoma Bank, Iowa city, Iowa, USA) as described previously [17, 18]. Apoptotic nuclei were identified by first labeling the tissue sections with fluorescein isothiocyanate (FITC) followed by labeling with terminal dUTP nick-end labeling (TUNEL) (11684795910; Roche Applied Science, Indianapolis, Ind, USA) and lamina, using methods described previously in our lab [19]. The nuclei were counterstained with 4′,6-diamidino-2-phenylindole (DAPI).

### 2.7. Exercise Training

Supervised strength training was conducted 3 times per week for 16 weeks. The exercises consisted of using Body Masters machines and free weights. The exercises included Monday/Friday: machines—leg press, bench press, pulldown, triceps extension, and calf raise. Dumbbell arm curls were performed with free weights. Wednesday: leg extension, leg curl, and seated rowing were performed on the weight machines while dumbbell shrug, dumbbell lateral raises, and dorsi flexion were conducted with free weights. The training intensity and volume load of each workout session were recorded in a training log. The training consisted of three weeks where intensity and load were increased by 5% per week followed by a one week where the volume load was decreased by 5%. A rating of perceived exertion (RPE) using a 6–20 scale was obtained from the subject after each exercise session.

### 2.8. Results

The subject had good compliance to strength training, as she completed 48 sessions in 16 weeks. There were no flare-ups of disease activity, training-related injuries, or any other adverse events during the 16 weeks of strength training, and there was no change in medication over the course of the study.

The physical and body compositional characteristics of the subject are given in Table 1. The subject's body weight and FFM increased by 3.7% and 2.1%, respectively, after strength training. Training-induced improvements were found in DAS28 (3.39 to 2.00) and pain VAS (40 mm to 10 mm); REE was 10.4% greater after strength training as compared to the pretraining level. There was a 12.6% improvement in the 50-foot walk test (9.54 s to 8.34 s) following strength training, but there was no change in 30-second chair stand test (15 reps) as compared to the pre training tests. VO_2_ peak was 8% greater after strength training as compared to pre training values (1.76 to 1.84,L/min). The treadmill exercise time to fatigue increased after strength training (15.30 to 19.30 min) as compared to the fatigue assessment before training (Table 1).

ESR and C-reactive protein were measured as indices of systemic inflammation, but both markers were withinnormal ranges before and after strength training (Table 2). C-reactive protein levels appeared to climb slightly, whereas ESR decreased after resistance training. Resistance training did not markedly alter other blood markers for cardiovascular risk in this subject. However, there was a trend for a slight increase in blood lipid levels after 16 weeks of exercise training in this subject.

There were a 13.6% and 9.8% increase in knee extensor peak torque and power, respectively, and a decrease in work fatigue from 18.4% before strength training to 15.6% after strength training (Table 2). Knee flexor peak torque and average power were 51.9% and 82.5% greater, respectively, after strength training. Knee flexor work to fatigue decreased from 27.8% to 13.4% (Table 3).

**Figure 1:**
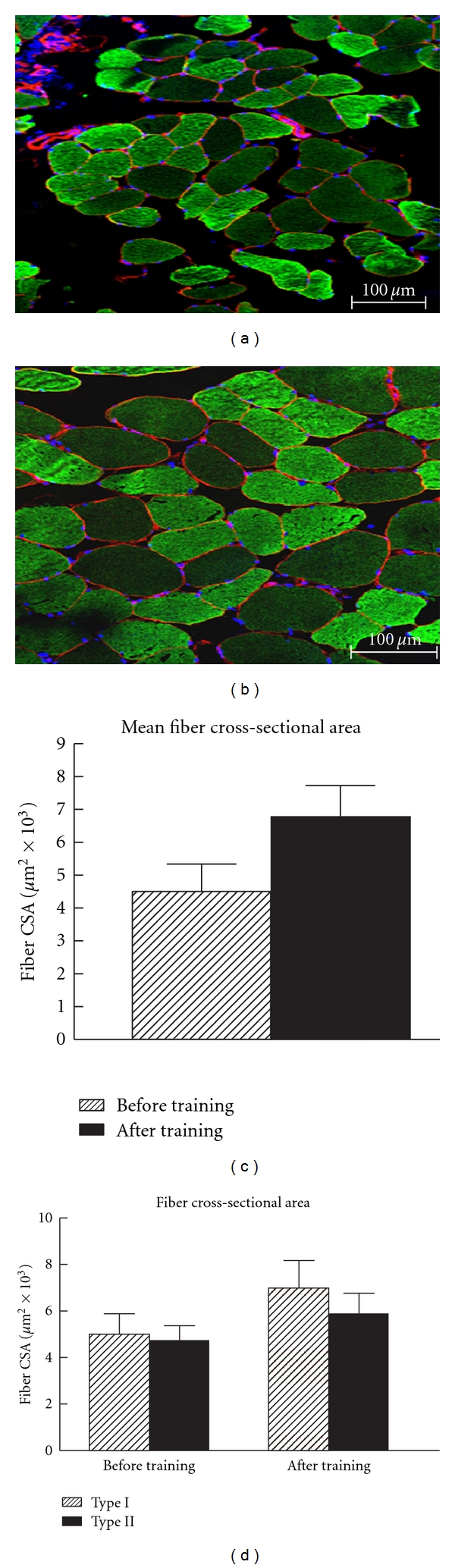
Frozen tissue cross sections incubated with anti-fast myosin heavy chain (F59). Fibers that were stained for FITC (green) were identified as type II fibers, and negatively stained fibers were identified as type I fibers. The basal lamina of each fiber was stained (red) with an anti-laminin antibody. (a) Type I and type II fibers from the vastus lateralis muscle obtained before exercise training. (b) Type I and type II fibers from the vastus lateralis muscle obtained after 16 weeks of strength training. (c) Quantification of the mean fiber area from the vastus lateralis. These data include both type I and type II fibers. The data were obtained before and after strength training. The data represent mean ± standard deviation of fiber area. (d) Type I and type II fiber areas of the vastus lateralis before and after strength training.

Strength training did not alter fiber type distribution in the vastus lateralis muscle from before training (type I, 32.6%, type II, 67.4%) to after strength training (type I, 39.7%; type II, 60.3%). Type I fiber CSA was slightly greater than type II (4997.9 *μ*m² versus 4430.1 *μ*m²) before training. The strength-training program increased total fiber CSA by 49.7% (4634.8 *μ*m² versus 6938.6 *μ*m²). This was the result of a 64.9% increase in type I fiber CSA (4998.0 *μ*m² to 8239.5 *μ*m²) and a 37.3% increase in type II fiber CSA (4430.1 to 6082.8 *μ*m²). Examples of tissue cross sections are shown in Figure 1.

**Figure 2:**
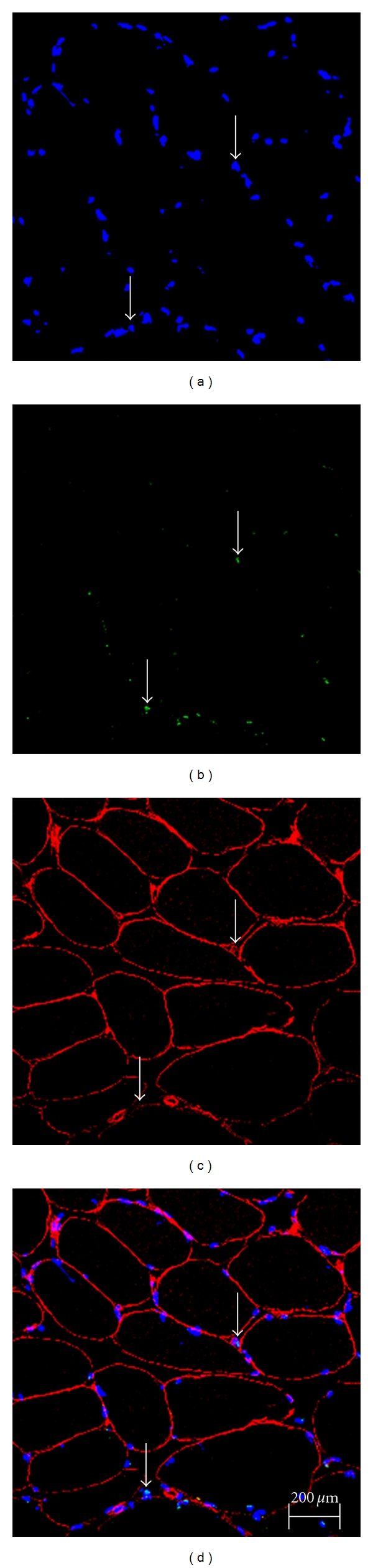
Examples of tissue cross-sections. (a) DAPI-stained nuclei. (b) Apoptotic nuclei as indicated by the TUNEL assay. (c) The basal lamina of fibers was identified by immunocytochemistry. (d) Overlay of DAPI, TUNEL, and lamina staining. The arrows indicate examples of apoptotic nuclei.

Apoptotic nuclei were present (Figure 2), but they made up a small percentage of the total myonuclei before strength training (1.9%). Apoptotic nuclei decreased modestly after strength training (1.6%). Average nucleus/fiber CSA increased slightly after strength training (from 0.08 to 0.12 nuclei/*μ*m²).

## 3. Discussion

In this paper, we show that resistance training improved walking performance, time to fatigue on a treadmill, and knee extensor and knee flexor isokinetic power and strength (torque) in a subject with RA. The improvement in function could be attributed to the increase in muscle fiber cross-sectional area and the accompanying modest increase in fat-free mass. Type I fibers appeared to be more responsive to strength training than type II fibers, in the vastus lateralis of this subject. Nevertheless, it is important to note that type II fibers are particularly susceptible to atrophy in rheumatoid cachexia [5] so, even though this fiber type did not enlarge as much as type I fibers, the type II fiber population still was capable of increasing in size with strength training in RA. In addition, type II fibers were more abundant in the vastus lateralis of this subject, so small changes in the size of this fiber type likely translated into important physiological changes and contributed to greater strength production at the whole muscle level.

It is not clear if the small decrease in nuclei undergoing apoptosis (as indicated by the TUNEL assay) had a significant role to play in improving muscle mass. However, there was an increase in nuclear density, suggesting that more nuclei (presumably satellite cells) were activated as a part of adaptations to strength training [20]. Nevertheless, even a very modest decrease in TUNEL-positive nuclei may have provided a means for accumulating a greater number of myonuclei. A greater total muscle nuclei population would have the potential for a greater transcription of mRNA and if translated, this would result in greater protein accumulation and muscle mass after resistance training. Nevertheless, the data indicate that resistance exercise improved muscle function and mass, and this is consistent with the idea that rheumatoid cachexia may be able to be reversed or at least slowed by an exercise intervention that includes resistance training, perhaps in part, by protection of activated satellite cells from apoptosis-induced cell death [9].

Strength training did not increase the symptoms associated with RA in the subject studied in this project. In contrast, the disease activity and pain score were reduced after 16 weeks of strength training as compared to before training. However, it is not clear if the reduction in the DAS index was a direct result of improving muscle structure and function or if it was the result of other conditions, such as an exercise-induced mediation of systemic oxidative stress.

Our results are consistent with reported improvements in exercise-associated elevations in strength and endurance training in RA patients [21]. Improvement in FFM was expected, but, in this subject, FM increased along with FFM. It is possible that the exercise program did not result in a net caloric deficit, perhaps because the subject increased her food intake over the course of the study. Alternatively, this may indicate that resistance training was unable to prevent the accumulation of body fat, which often accompanies RA [1, 2]. In fact, persons with RA have a greater likelihood of obtaining cardiovascular disease [22, 23]. An increase in total body fat mass, and poor blood lipid profiles are risk factors for cardiovascular disease, and these negative changes have been implicated in the progression of RA [1, 2]. The subject examined in the present study had a trend towards greater adiposity (increased FM) and a trend towards slight increases in the lipid profile for triglycerides and cholesterol after 16 weeks of exercise intervention. This suggests that resistance training was unable to lower cardiovascular risk as indicated by blood lipid profile, and this may be indicative of a potential progression towards metabolic syndrome.

It has been proposed that there may be a link between increased adiposity/metabolic syndrome [24] and cardiovascular disease [25, 26] in RA patients, perhaps triggered by elevated oxidative stress or inflammation. An elevation in systemic inflammation as indicated by increased C-reactive protein levels has been frequently noted in RA [27]. However, this is not a universal observation because recent data suggest that, for some RA patients, C-reactive protein may be inactive (<1 mg/dL), yet radiographic progression and joint swelling may be evident [28]. In addition, some RA patients with high body fat and metabolic syndrome do not have elevations in C-reactive protein levels [26], a systemic marker for inflammation. Nevertheless, if we assume that in most cases C-reactive protein is a highly sensitive marker for inflammation, then increases in this marker could point towards progression of the disease. C-reactive protein levels were quite low before exercise training in the subject that is described in this study, and therefore she may fit in with the group of patients where C-reactive protein is a less responsive indicator of RA [26]. Nevertheless, it was interesting to note that resistance training did not improve this marker and may have even pushed it to slightly higher levels. Several possibilities could explain this finding. For example, it is potentially possible that resistance training resulted in a slight increase in systemic inflammation and a corresponding slight increase in C-reactive protein. However, if that was the case, we would also have expected ESR to increase after 16 weeks of exercise training, but it did not. An alternative explanation is that the progression of elevated adiposity (due to diet or disease progression) might have contributed to the slightly higher C-reactive protein levels. In either scenario, in this study, we are unable to rule out that resistance training alone might contribute to, and/or fails to prevent, elevated cardiovascular risk in persons with RA.

It is likely that if exercise training had involved a combination of resistance and endurance types of exercise, then the lipid profile along with C-reactive protein may have improved. While adding an aerobic component to the exercise program may have reduced FM, and perhaps increased VO_2_ peak more than strength training alone, it is not known if combining strength and endurance exercise would be beneficial for reducing rheumatoid cachexia, muscle function, and measures of pain in RA as compared with strength training alone.

Although RA has been associated with muscle loss, the data in this case study suggest that improvements in isokinetic strength and muscle hypertrophy are possible in a subject with RA. Our data suggest that proper exercise training that includes strength training may help to reverse or reduce muscle loss in RA patients. Muscle apoptotic signaling does not increase with strength training in RA subjects, and therefore exercise appears to provide therapeutic anabolic effect for muscles of RA patients. Rather, similar to exercise-induced reductions in apoptotic signaling that have been shown in experimental animals [9, 17, 29], the data in this case study suggest that strength training is a safe and maybe an effective tool for reducing disease activity and pain and restoring physical function, muscle mass, muscle fiber cross-sectional area, and muscle strength in a patient with stable RA. Additional work is needed to determine if adding aerobic training to strength training will provide improvements in systemic inflammation and the blood lipid profile, reduce fat mass, increase muscle function, and reduce rheumatoid cachexia in a similar fashion or better than that which is seen with strength training alone.

## Figures and Tables

**Table 1:** Physical characteristics.

	Before training	After training
Body weight (kg)	71	73.6
BMI (kg/m^2^)	27.7	28.7
Fat (%)	29.9	30.9
Fat mass (kg)	21.23	22.74
Fat free mass (kg)	49.8	50.9
REE (kcal/D)	1339	1479
DAS 28 (0–28)	3.39	2
Pain (VAS 0–100 mm)	40	10
50 feet walk (s)	9.54	8.34
30 Sec CST (reps)	15	15
VO_2peak_ (L/min)	1.76	1.84

BMI: body mass index; REE: resting energy expenditure; DAS28: disease activity score; VO_2 peak_: peak oxygen consumption.

**Table 2:** Blood markers for inflammation and lipids.

	Before training	After training
ESR (mm/hr)	2	1
C-reactive protein (mg/dL)	0.038	0.111
Triglycerides (mg/dL)	53	74
Cholesterol (mg/dL)	182	197
HDL (mg/dL)	56	61
LDL (mg/dL)	115	121
VLDL (mg/dL)	11	15

ESR: erythrocyte sedimentation rate; HDL: high-density lipoproteins; LDL: low-density lipoproteins; VLDL: very-low density lipoproteins.

**Table 3:** Muscle performance.

	Before training		After training	
	Extension	Flexion	Extension	Flexion
Peak torque (Nm)	116.7	28.4	132.8	43.2
Peak torque/BW (%)	55.1	13.4	62.8	20.4
Work/BW (%)	54.0	14.0	64.0	22.5
Work fatigue (%)	18.4	27.8	15.6	13.4
Average power (Watts)	113.0	24.0	124.1	43.8

BW: body weight; Newton-meters: Nm.
